# Progress in Genito-Urinary Surgery

**Published:** 1903-03-21

**Authors:** 


### PROGRESS IN GENITO-URINARY SURGERY.

(*Concluded from page* 411.)

**Tubercular Disease of the Prostate.**—Crandon, of Boston, Mass.,[5] considers that this disease is much more frequent than its diagnosis. It may arise primarily or by descending or ascending infection. The writer describes a specimen he possesses of the primary disease, obtained from a case in which no other tubercular deposit could be found in the body. He also adduces an example of descending infection. The patient, who had previously Addison's disease, developed symptoms like those of senile enlarged prostate. At the post-mortem the left supra-renal capsule, the left kidney and the prostate were found to be tubercular. The oldest deposits were those on the suprarenal capsule. The Addison's disease had been of three years' duration. The prostate was most affected on the left side, and from that side the disease appeared to have spread to the other side. The left ureter was diseased near the bladder, but the testicles, epididymes, and vasa-deferentia were healthy. The symptoms of prostatic tuberculosis are those of chronic prostatitis, frequent urination, burning pain with micturition, and the passage of a few drops of blood before micturition. The gland becomes moderately enlarged, soft, and tender, especially in the posterior part. Malignant tumour and stone in the bladder have to be excluded in the diagnosis. M. Möller[6] has placed on record a case of primary tuberculosis of the prostate which followed a traumatic prostatic abscess. Suppurative prostatitis of traumatic origin is very rare, and primary tuberculosis is also rare. Two years before Möller saw him the patient had had a heavy fall from a tramway car on to the pavement. He had been confined to bed for three weeks after this, having much pain in the buttocks. By that time a large abscess in connection with the prostate had formed, and from this a large quantity of pus was evacuated. Two years later he complained of pains in the lower part of the pelvis, and after a few months died of general tuberculosis. A post-mortem showed the starting point of the tuberculosis to have been a caseous mass in the prostate. The rest of the genito-urinary apparatus was healthy.

**Removal of the Bladder and Prostate for Carcinoma.**—Malcolm M. Harris, of Chicago,[7] records a very remarkable case in which he performed successfully the above operation. The patient was fifty-three years old. About a year previously he accidentally discovered blood in the urine. This recurred, and bladder irritability developed and loss of flesh and strength were noticeable by the end of the year. Upon rectal examination the prostate was found to be enlarged, firm, irregular and tender. The cystoscope revealed a bleeding mass involving the trigonum and extending beyond the ureteral openings. Suprapubic cystotomy was performed and the growth, a carcinoma, was found to involve the whole of the trigonum and to extend a little above both ureteral orifices, and it was obvious also that the prostate was involved and that it would be necessary in order to get the growth away to remove the prostate and base of the bladder *en masse*. The bladder was freed by blunt dissection on each side as far down as the base, the urethra divided close to the triangular ligament, and beginning at this point and working backward and upward the prostate and bladder were separated from the rectum. The considerable hæmorrhage was lessened by keeping the bladder well drawn forward. The ureters as they appeared were divided beyond the disease. A few enlarged lymph glands by the side of the bladder were removed. A portion of the vertex of the bladder was left, and the ureters were drawn through small slits in this and fixed there by sutures. Then this remnant of the bladder was stitched by its edge to the inner edge of the suprapubic opening except below. The cavity in the pelvis was packed with gauze and a large drainage tube inserted. The peritoneum was not opened. In two weeks the patient was able to sit up. In about a month he was up and about. The ureteral openings could be seen in the small bladder. A catheter passed through the urethra reached the bladder and was retained permanently. At the end of two months the patient caught cold when out of doors, and in a few days died of pneumonia. A tongue-shaped process lined with epithelium was found extending from the bladder almost to the end of the urethra. According to Wendel the immediate mortality, after complete extirpation of the bladder, is 60 per cent. (10 cases, six deaths). After partial extirpation, 24·5 per cent. (57 cases, 14 deaths). Shock and uræmia are the chief causes of death. The implantation of the ureters into the bowel is almost unjustifiable. Therefore the plan above adopted is to be recommended in males. The bladder recuperates so remarkably that even the smallest portion saved may develop into a useful receptacle for urine. As another example of this recuperative power, the author mentions the case of a cyclist who ruptured his posterior urethra, and had extravasation of urine and sloughing of the greater part of the bladder. The major part of the bladder, with the exception of the trigonum and a bit of the posterior wall was therefore removed. After a time the patient could hold his urine five or six hours, and go all night without difficulty.

[5] Vide Abst. Centralb. f. Chir., Oct. 11, 1902, p. 1078. [6] Vide Abst. Ibid., Sept. 6, 1902, p. 957. [7] Annals of Surgery, Oct., 1902, p. 509.

